# Exercise and Childhood Cancer—A Historical Review

**DOI:** 10.3390/cancers14010082

**Published:** 2021-12-24

**Authors:** Javier S. Morales, Pedro L. Valenzuela, Daniel Velázquez-Díaz, Adrián Castillo-García, David Jiménez-Pavón, Alejandro Lucia, Carmen Fiuza-Luces

**Affiliations:** 1 MOVE-IT Research Group, Department of Physical Education, Faculty of Education Sciences, Universidad de Cádiz, 11519 Cadiz, Spain; javier.salvador@uca.es (J.S.M.); daniel.velazquez@uca.es (D.V.-D.); david.jimenez@uca.es (D.J.-P.); 2 Biomedical Research and Innovation Institute of Cádiz (INiBICA) Research Unit, Puerta del Mar University Hospital, University of Cádiz, 11009 Cadiz, Spain; 3 Faculty of Sport Sciences, Universidad Europea de Madrid, 28670 Madrid, Spain; pedroluis.valenzuela@universidadeuropea.es (P.L.V.); alejandro.lucia@universidadeuropea.es (A.L.); 4 Physical Activity and Health Research Group (‘PaHerg’), Research Institute of the Hospital 12 de Octubre (‘imas12’), 28041 Madrid, Spain; 5 Fissac—Physiology, Health and Physical Activity, 28015 Madrid, Spain; adrian@fissac.com; 6 CIBER of Frailty and Healthy Aging (CIBERFES), 28029 Madrid, Spain

**Keywords:** physical activity, survival, stem cell transplantation, leukemia, solid tumors, exercise is medicine

## Abstract

**Simple Summary:**

Childhood cancer survivors are at risk of developing important adverse effects, but there is growing evidence that physical exercise could help in this regard. The present review summarizes the history of pediatric exercise oncology and the main milestones achieved along the way. Overall, physical exercise appears to be safe and beneficial even during the most aggressive phases of pediatric cancer treatment and can represent an effective coadjuvant therapy for attenuating cancer-related adverse effects.

**Abstract:**

Childhood cancer survivors are at risk of developing important adverse effects, many of which persist for years after the end of treatment. The implementation of interventions aiming at attenuating tumor/treatment-associated adverse effects is therefore a major issue in pediatric oncology, and there is growing evidence that physical exercise could help in this regard. The present review aims to summarize the main milestones achieved in pediatric exercise oncology. For this purpose, we conducted a systematic review of relevant studies written in English in the electronic database PubMed (from inception to 14 August 2021). This review traces the field of pediatric exercise oncology throughout recent history based on three fundamental pillars: (i) exercise during childhood cancer treatment; (ii) exercise during/after hematopoietic stem cell transplantation; and (iii) exercise after childhood cancer treatment. Accumulating evidence––although still preliminary in many cases––supports the safety and potential benefits of regular exercise (with no major contraindications in general) in the childhood cancer continuum, even during the most aggressive phases of treatment. Exercise can indeed represent an effective coadjuvant therapy for attenuating cancer-related adverse effects.

## 1. Introduction

Medical advances in the management of childhood cancer have led to an unprecedented increase in the overall 5-year survival rate, which currently averages 80–85% [1,2]. However, childhood cancer survivors (CCS) are still at high risk of adverse effects, many of which might persist years after treatment [3,4]. Notably, CCS might show an impaired left ventricular (LV) function and a higher waist-to-hip ratio for decades compared to their peers who did not have cancer during childhood [3]. Cancer and its treatments are usually associated with pain, limitations in range of motion, deficits in balance, neuropathic symptoms and gait impairment, alterations in body composition (excess adiposity, especially at the central/visceral level, and reduced muscle mass), as well as an increased risk of overweight/obesity, low bone mass, and mental problems [5,6,7,8,9,10,11,12,13,14]. Approximately two out of three CCS present with at least one chronic condition earlier in life than the general population, on average ~17.5 years after cancer diagnosis [4]. Another cancer-related adverse effect is fatigue, which contributes to the sedentary behavior and low physical activity (PA) levels that are often found in CCS [15,16,17,18]. Indeed, CCS frequently engage in health-risk behaviors, including excess screen time [19,20]. In part because of this [21,22,23], the fitness status of CCS is typically impaired during and after treatment [21,24,25,26], as reflected by low values of cardiorespiratory fitness (CRF) and muscle strength, or by an impaired ability to perform activities of daily living [27]. This is of clinical relevance because CRF and muscle strength are important health markers in children and adolescents [28,29].

For the above reasons, the implementation of interventions aiming at attenuating cancer-related adverse effects is a major issue in pediatric oncology. In this regard, there is growing evidence that physical exercise is safe both during the acute and off phase of treatment [30], and can also help to reduce cancer treatment-related side effects in CCS [31,32]. Notably, physical exercise interventions (with the vast majority combining aerobic and resistance training) during and/or after treatment have been shown to attenuate several of the harmful effects associated with medical therapies for a wide variety of pediatric tumors. Notably, there can be: a decrease in the number of infections after allogenic hematopoietic stem cell transplantation (HSCT) for the treatment of blood cancer [33]; the preservation of LV function in children receiving neoadjuvant treatment for solid tumors or intensive chemotherapy treatment for leukemias [34], as well as in children with acute lymphoblastic leukemia (ALL) [35,36]; increases in CRF levels in children with ALL [37,38,39,40] or with different types of blood cancers [41,42] or malignancies [43,44,45,46,47], as well as in survivors of brain tumors [48]; improvements in lean mass in children with all types of cancers [49] and in muscle strength in children with solid tumors undergoing neoadjuvant therapy [17], as well as in children with ALL [37,39,50] or with different types of cancer [45,46,47,49,51]; mild improvements in immune function in the context of HSCT [52,53]; improvements in cognitive function in survivors of brain tumors [48] and rises in PA levels in children with ALL [38] or with different types of cancer [45] as well as with bone tumors [54]; improvements in the health-related quality of life (HRQoL) in children with blood cancer [41]; decreases in waist circumference, waist-to-hip ratio, and percentage of fat mass in long-term survivors of childhood ALL [35]; and decreased pain [55] and fatigue in children with different types of cancer [45].

However, scarce data are available on the biological mechanisms explaining the potential benefits of physical exercise during the pediatric cancer continuum. In any case, the biological effects of exercise in the context of pediatric cancer are not likely to differ essentially from those reported in children in general. On the one hand, regular exercise, especially if combining aerobic and resistance modalities, has a beneficial effect on the chain of interactive events between the central nervous system and the contraction of the skeletal muscles that are involved in most types of exercises or physical activities [56]. These events include blood oxygenation (which depends on pulmonary function), the supply of oxygenated blood to the working muscles (which depends on LV function and blood oxygen transport capacity), and the ability of muscles to consume oxygen and to produce force while contracting. As a result, regular exercise has the potential to increase CRF and attenuate, at least partly, some of the main detrimental effects of treatment, such as lung fibrosis and pulmonary impairment caused by radiotherapy and a decline in cardiac function (i.e., cardiotoxicity) induced by radiotherapy and chemotherapy (particularly, but not only, anthracyclines). Exercise can also attenuate decreases in blood oxygen transport capacity (i.e., treatment-induced anemia) and decreases in muscle mass, and thus in muscle strength (induced by the direct catabolic effects corticosteroids or immunosuppressant drugs or indirectly by gastrointestinal toxicities leading to malnutrition and catabolism) [56]. On the other hand, many of the multisystemic benefits of exercise are potentially attributable to the release of muscle-derived factors, collectively known as ‘myokines’ (or ‘exerkines’ if released from non-muscle tissue during exertion) [57]. These molecules produced in the exercise milieu can have a local muscle anabolic effect or travel through the bloodstream to reach different tissues where they induce numerous beneficial effects, including among others, an anti-inflammatory action, a thermogenic/lipolytic effect, or the promotion of neurotropism in the central nervous system. Regarding the latter, exercise training has been shown to have a beneficial impact on brain structure in children with brain tumors treated with radiation [58].

**Figure 1:**
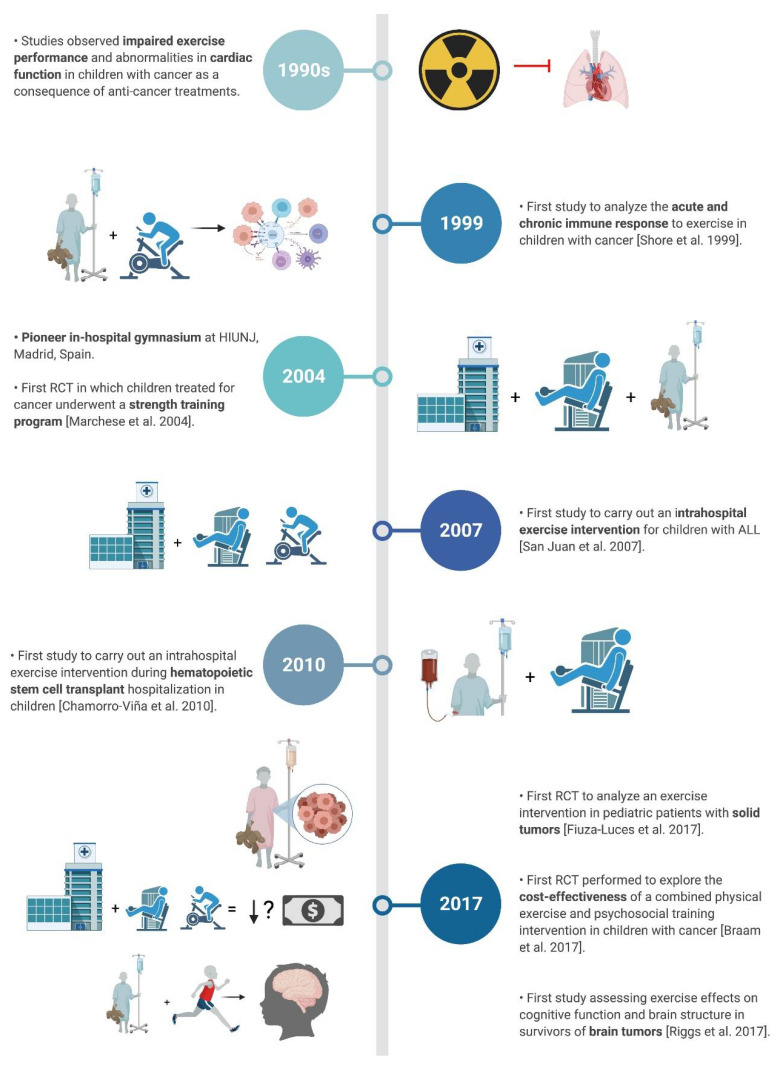
Distribution of articles according to the main milestones that contributed to knowledge on the childhood cancer and physical exercise binomial. Abbreviations: ALL, acute lymphoblastic leukemia; HIUNJ, Hospital Infantil Universitario Niño Jesús; RCT, randomized controlled trial.

The purpose of this narrative, non-systematic review, was to summarize through an analysis of the temporal progress, the main milestones in the field of exercise and pediatric oncology (Figure 1) since the publication of the first studies on the topic. Two authors (DVD, JSM) conducted a search to identify relevant studies written in English in the electronic database PubMed (from inception to 14 August 2021) using the following search strategy: (child* OR pediatric OR infant OR adolescen*) AND (exercise OR physical activity OR training) AND (cancer OR carcinoma OR tumor OR tumour OR neoplasm OR maligna* OR leukemia OR leukaemia OR oncology). Studies were first screened by title and abstract, and the full text of those studies that seemed to address the topic of this review was retrieved. We focused mainly on studies conducted with CCS who were performing an exercise training intervention during and/or after treatment, and studies representing a major milestone contributing to the knowledge on childhood cancer and physical exercise (particularly, based on novelty or design). We also conducted a search in the clinicaltrials.gov (accessed on 12 December 2021) registry to identify ongoing trials that could be published in the foreseeable future.

The fact that childhood cancer is a rare disease [59] might explain why research in the field of pediatric exercise oncology is not as prolific as in adult cancer, with recruitment of large patient cohorts being comparatively much more challenging in the former. In fact, when conducting a similar systematic search in PubMed for cancer and exercise during either childhood/adolescence or adulthood, respectively, the difference in the number of hits is overwhelming: 2489 results for the former (using the same systematic strategy as for the present review), vs. 12,041 for adults and older adults (using the terms: (adult* OR older OR elderly OR men OR women OR senior OR geriatric) AND (exercise OR physical activity OR training) AND (cancer OR carcinoma OR tumor OR tumour OR neoplasm OR maligna* OR leukemia OR leukaemia OR oncology)). This review traces the field of pediatric exercise oncology throughout history on three fundamental pillars: (i) treatment, (ii) HSCT, and (iii) post-treatment.

## 2. Exercise during Childhood Cancer Treatment

Several studies [60,61,62,63,64,65,66,67,68,69] in the early 1990s found an impaired exercise performance together with cardiac function alterations as a consequence of anti-cancer treatments (notably, chest irradiation and anthracycline treatment). A study published in 1993 [70] showed for the first time that the muscle strength of CCS might be impaired for many years after therapy for childhood leukemia. Based on this earlier finding, several authors suggested that physical exercise could help to preserve the health status of this population [63,64,65,68,69,70]. Information on relevant studies analyzing the effects of exercise intervention on CCS during treatment is summarized in Table 1.

The first study showing a positive association between leisure-time PA during cancer and health benefits—such as an improved psychosocial well-being—in adolescents was published in 1999 [71]. It was also in the same year that the effects of an individually-planned 12-week exercise intervention in adolescents undergoing treatment for different types of childhood cancer (including ALL) were first studied [43]. The authors (Shore and Shepard) found that exercise improved different health markers, such as peak oxygen uptake (VO_2peak_) and mental health. It must be noted that exercise resulted in an impaired immune function, although this type of effect was also observed in a group of healthy children—albeit to a lesser extent compared to patients—and the authors stated that the impairment in patients’ immune function was ‘not clinically relevant’ [43]. In light of a hypothetical immunosuppressant effect of vigorous exercise and of the fact that immune function is often affected in pediatric cancer patients, Ladha et al. analyzed the effects of acute exercise on the neutrophil count and function in children and adolescents who were receiving maintenance treatment for ALL [72]. The authors showed that an acute bout of moderate–vigorous intensity exercise (70–85% VO_2peak_) did not elicit any type of negative neutrophil response among CCS, and their response was actually similar to that of healthy children [72].

Another pioneer study was conducted by Marchese et al., who in 2004 published the first randomized controlled trial (RCT) with children (aged 4 to 11 years) receiving maintenance therapy for ALL, where the intervention group performed a home-based muscle strength training program [50]. Four months after the intervention, patients showed improvements in ankle dorsiflexion active range of motion and leg muscle (knee extensors) strength [50]. In 2005, in view of the strong evidence supporting the benefits of PA in healthy children [73,74,75,76,77,78,79,80,81], a narrative review suggested that performing PA might also attenuate bone loss and the risk of obesity in children with ALL [82].

Given the emerging evidence for potential exercise-associated benefits in children with cancer at that time, the Pediatric Oncohematology Department at the *Hospital Infantil Universitario Niño Jesús* (HIUNJ, Madrid, Spain) set up an in-hospital gymnasium in 2004. The gym was equipped with cycle ergometers and weight training machines that were specifically designed for the body dimensions of children. This milestone was presented in the journal *Leukemia* as a complementary treatment tool against childhood cancer [83], and paved the way for subsequent studies assessing the effects of supervised exercise in very young children (aged 4–7 years) during the maintenance phase of treatment against ALL [37,84,85]. The main findings of these studies were that supervised in-hospital exercise intervention (combining strength and aerobic exercises, with a special focus on the former) improves CRF, muscle strength and functional mobility in this patient population [37,84]. On the other hand, this type of intervention was not associated with any major adverse effect and elicited no changes in circulating levels of insulin-like growth factor (IGF)-1 (a survival agent and growth factor that plays a key role in the antiapoptotic and mitogenic pathway in many cell types) and IGF-2 or IGF-binding proteins, thereby suggesting that exercise can be safely undergone during treatment [85].

The abovementioned studies and others published in later years focused on hematological tumors, mainly ALL. Between 2009 and 2018, several randomized controlled trials (RCT) were published with the aim of further delving into the effects of exercise interventions in pediatric patients at different stages of ALL treatment [38,39,86,87]. Thereafter, two RCTs were published that aimed at exploring the cost-effectiveness and the effects on HRQoL of a combined physical exercise and psychosocial intervention during (or within a year after) treatment in a cohort of 68 Dutch patients with different types of childhood cancers [55,88]. However, the authors did not report any major benefit after the intervention [55,88]. Of note, is the fact that the postintervention assessment was performed one month after the end of the intervention and could have potentially accounted for a certain “detraining effect”, thereby explaining, at least partly, the lack of major intervention-induced benefits. Increasing evidence on the effects of exercise in the context of childhood cancer led to the publication in 2013 of a Cochrane review [89] (which was subsequently updated in 2016 [90]) including several of the abovementioned RCTs [38,39,50,86]. In the most recent update, Braam et al. reviewed six studies (five RCTs and one clinical controlled trial (CCT)) evaluating the effects of exercise intervention in a total 171 children/adolescents aged <19 years, all being treated for childhood ALL [90]. The authors found significant benefits in markers of CRF, bone mineral density, and muscle strength.

In general, the evidence in the field of exercise intervention is much more limited for pediatric solid tumors compared to leukemias. In 2013, Winter et al. conducted the first CCT implementing an exercise intervention in patients with pediatric bone tumors [54]. Despite the fact that patients had a malignant bone tumor in a lower extremity and were therefore at high risk of having a functional limitation and low PA levels, the results showed that exercise was feasible and beneficial even in this population [54]. In 2017, the first two RCTs—derived from the ‘Physical Activity in Pediatric Cancer’ (PAPEC) trial—were published, assessing the effects of an in-hospital exercise intervention (lasting for the whole treatment with neoadjuvant chemotherapy) in children/adolescents with solid tumors [91,92]. The main finding was an increase in muscle strength in the intervention arm only [91]. However, no between-group differences were found for secondary outcomes such as CRF, functional capacity, PA levels, or HRQoL. Of note, the exercise intervention proved to be safe, and did not affect the blood inflammatory profile or immune cell counts despite the aggressive, immunosuppressive nature of anticancer treatments against this type of tumor [92]. As part of the PAPEC trial, our research group performed an ancillary analysis investigating for the first time the individual responsiveness to a supervised in-hospital exercise intervention performed during pediatric cancer treatment [93]. Although most (≥80%) children showed improvements in muscle strength after the intervention, a considerable interindividual variability was found for the improvements in functional mobility and CRF (with an average prevalence of nonresponders of ≥50%, and even more among those with a higher fitness status at baseline) [93].

Given the number of RCTs analyzing the effects of exercise in the context of ALL or other pediatric cancers published in the last few years, our research group performed a systematic review and meta-analysis on the effects of exercise intervention for patients with any type of childhood cancer during treatment, which was published in 2018 [31]. After analyzing eight RCTs (total *N* = 283 patients) we found that exercise intervention during childhood cancer treatment does not affect mortality or relapse risk and in turn improves functional mobility, as assessed with the ‘Timed Up and Down Stairs’ test [31].

More recently, we published a prospective cohort (*N* = 169 children with a new diagnosis of cancer) study that assessed the effects of supervised in-hospital exercise intervention on major clinical endpoints (notably, risk of mortality, as well as metastasis and disease relapse, hospitalization days, and cardiovascular function) during active treatment for basically any type of childhood cancer (median duration of the intervention ~22 weeks) [34]. The cohort was followed from the start of treatment (i.e., neoadjuvant (for solid tumors) or intensive chemotherapy (for leukemias)) for up to five years. The results indicated that the exercise intervention was safe and benefited echocardiography-determined LV function in the short term, yet this cardioprotective effect was not maintained after one year [34]. Another important finding was that the exercise intervention reduced the total number of hospitalization days, with subsequent benefits not only for the wellbeing of the patients, but also in terms of the economic burden [34].

Finally, three trials have been published in the last two years (2020 and 2021), evaluating the effects of a combined (muscle strength and aerobic exercise) training intervention in children and adolescents undergoing treatment for different types of cancer [44,45,46]. Nielsen et al. applied a supervised in-hospital exercise intervention that included for the first time two healthy classmates chosen as ‘ambassadors’, with the aim of improving patients’ motivation for engaging in the exercise intervention [44]. This novel multicomponent intervention combining an exercise training program and the support of the ambassadors resulted in a high adherence rate as well as in physical function improvements, mitigating the alterations in CRF that are usually experienced by children throughout cancer treatment [44]. In the MUCKI (informal term for ‘muscle’ in German) RCT by Stössel et al., childhood cancer patients undergoing intensive cancer treatment performed a moderate-intensity exercise intervention in inpatient and outpatient clinics as well as at home (during outpatient stays) [45]. The authors found a significant beneficial effect of exercise on leg (but not on arm) muscle strength, walking performance, fatigue, self-esteem, and self-reported physical function, although no benefits were found for anthropometric outcomes or for the remainder of the HRQoL domains [45]. In 2021, Saultier et al. implemented a six-month exercise intervention including both in-hospital and outdoor activities [46]. The intervention induced improvements in CRF and HRQoL and these benefits remained six months later [46].

### Ongoing Studies

We identified an ongoing CCT that started in April 2017 and aims to enroll 380 children and adolescents with hemopathies (malignant and nonmalignant conditions) undergoing conventional treatment and/or HSCT (NCT04090268). In this trial, which is expected to be completed in April 2022, participants in the intervention group are being assigned to a 12-week precision exercise program. Oxidative metabolism, muscle strength and HRQoL are the main outcomes. Another ongoing trial is the IMPACT program, a CCT assessing the effects of a tailored exercise intervention (including a combination of aerobic, strength, balance, and flexibility exercises) which started in August 2021 and aims to enroll 250 children and adolescents receiving treatment for cancer or blood diseases (NCT04956133; estimated date of study completion, June 2028). A multicenter RCT that started in October 2014 and is being led by The Hospital for Sick Children (NCT02134782; Toronto, ON, Canada) aims to determine the effects of a 3-week individualized yoga intervention on fatigue, HRQoL and the use of systemic opioids, in children and adolescents (aged 8–18 years) receiving intensive chemotherapy for cancer or undergoing HSCT. This study is expected to be completed in October 2023.

## 3. Exercise during/after Childhood Hematopoietic Stem Cell Transplantation

HSCT is the treatment of choice for a number of malignant and nonmalignant conditions [94]. There has been a progressive increase in the number of patients undergoing this treatment modality over the last decades, particularly among children [95]. However, despite the advances in HSCT therapy, CCS treated with HSCT have a higher rate of severe, disabling, or life-threatening chronic conditions than those treated with conventional therapy (81% vs. 69%, respectively) [96]. As a result, there is a growing number of childhood-HSCT survivors, but also of post-treatment morbidities. Another concern is that HSCT and HSCT-related burdens can impair physical function. A recent systematic review and meta-analysis showed that years after treatment had ended, childhood HSCT survivors have an impaired CRF compared to non-HSCT controls (with a similar, albeit less clear trend for muscle strength and physical performance) [97]. There is also meta-analytical evidence that exercise is a safe strategy that can potentially preserve functional mobility, CRF and HRQoL in children and adolescents undergoing HSCT [98,99]. Therefore, there seems to be no reason to avoid physical exercise during childhood HSCT [100]. Table 2 shows a summary of the information regarding relevant studies on the effects of exercise intervention in pediatric cancer patients undergoing HSCT.

A study conducted at HIUNJ reported for the first time in 2008 that a tailored exercise intervention combining aerobic and strength exercises positively affected VO_2peak_, muscle strength, functional mobility and HRQoL in the short-term (i.e., within the first 12 months post-transplant) in childhood HSCT survivors who had suffered ALL or myeloid leukemia [41]. A subsequent study in the same hospital assessed the effect of an in-hospital exercise intervention lasting from the beginning of the conditioning phase until the end of the neutropenic phase (i.e., a mean of ~3 weeks) in pediatric cancer patients undergoing HSCT [52]. This intervention proved to be safe (with no negative effects on immune cell recovery) and prevented loss of body mass compared with nonexercised controls [52].

Subsequently, two noncontrolled studies [101,102] and one CCT [103] were published analyzing the effects of an in-hospital exercise intervention throughout the HSCT process in children and adolescents with malignant and nonmalignant conditions. Rosenhagen et al. [101] and Bogg et al. [102] found that exercise prevented loss of muscular strength—although no effect was found for HRQoL and in fact CRF and balance decreased [101,102]. On the other hand, Yildiz Kabak et al. [103] found that exercise improved muscle strength, CRF and functional mobility in children and adolescents undergoing HSCT, yet it induced no benefit overall on functional independence.

In a pilot RCT, we reported preliminary evidence that the combination of supervised and home-based exercise might be beneficial for the immune system—through increases in the mean ratio of CD56^dim^ cells and in natural killer cell cytotoxicity (or ‘killing capacity’)—of childhood HSCT survivors [53]. Thereafter, two RCTs were published as part of the ‘exercise therapy in pediatric stem cell transplantation’ trial (BISON study) [51,104], which examined exercise effects in pediatric patients throughout HSCT. The authors found that a tailored exercise intervention attenuated HSCT-related physical function decline [51,104], particularly among those who were unfit prior to the HSCT [104].

More recently, we studied the effects of an in-hospital exercise intervention throughout HSCT in a large cohort of children and adolescents (*N* = 65 and 53 in the exercise and control arm, respectively) who were followed from the beginning of the conditioning phase (for either autologous or allogeneic (allo)-HSCT) up to 6 years [33]. The intervention was safe and although it induced no overall effects—either beneficial or harmful—on major clinical endpoints (such as risk of mortality or graft-versus-host disease) it did reduce the number of total and viral infections after allo-HSCT [33]. Finally, Davis et al. investigated the effects of a 6-month intervention combining strength and aerobic exercises on the cardiometabolic profile and HRQoL of childhood HSCT survivors who had received total body irradiation (TBI; mean time since HSCT/TBI, 8 years) [42]. The results revealed an improvement in CRF, insulin resistance and some parameters of HRQoL, albeit with no changes in body composition [42].

### Ongoing Studies

Two of the aforementioned studies (NCT04090268 and NCT02134782) involve patients undergoing HSCT.

## 4. Exercise after Childhood Cancer Treatment

As previously mentioned, long-term cancer-related side effects are prevalent among CCS and can affect the fitness status of this population. This is of clinical relevance because previously identified risk factors for mortality in this population include self-reported inactivity and exercise intolerance (defined as VO_2peak_ levels below 85% of the age- and gender-predicted values) [105,106]. In this regard, despite the evidence that exercise intervention during treatment can attenuate these complications, it should be noted that in one of our studies [34], the exercise-induced benefits on LV function did not last more than one year after the start of treatment, which suggests that ideally, exercise should be maintained not only during the whole duration of treatment, but also after the end of it.

In 1993, Sharkey et al. reported the effects of a 12-week cardiac rehabilitation intervention (aerobic exercise) in a group of CCS (mean age at diagnosis and at the time of the study of 8 ± 4 and 19 ± 3 years, respectively) [107]. The authors found an improved time to exhaustion during a maximal exercise test for all participants compared to baseline, while the rest of the study outcomes remained unchanged [107]. Several years later, Takken et al. assessed the feasibility and efficacy of a 12-week exercise intervention (combining aerobic and strength exercises) performed between 12 and 36 months after the end of chemotherapy in CCS (ALL) [108]. Although no changes were found in any of the study outcomes (such as CRF, fatigue, muscle strength, or functional mobility), some problems related to the intervention, which was defined by the participants as ‘boring’ (with many of them in fact not completing the program), should be taken into account [108]. In addition, three of four girls dropped out of the study because they found the training sessions too intensive and hard to combine with their other activities. These issues might explain, at least partly, why no benefits were found with this intervention.

Järvelä et al. published a series of studies in which for the first time a home-based exercise intervention was applied in long-term CCS (ALL, >10 years from diagnosis) [35,36,109]. The authors observed an improvement in markers of cardiometabolic health (e.g., reduction in diastolic blood pressure, plasma insulin levels and insulin resistance, and improvements in vascular endothelial function and structure) and physical fitness (e.g., increase in CRF and muscle strength) in this population [35,36,109].

The accumulating evidence for the potential cardioprotective role of exercise intervention both during and after cancer treatment led to the publication of two recent meta-analyses on the topic [32,110]. Both included studies in pediatric populations that were receiving (or had completed) cancer treatment, with Bourdon et al. focusing specifically on the effect of aerobic exercise on CRF [110]. The two meta-analyses reported increases in CRF as assessed through a maximal [110] or submaximal test (e.g., the 6-min walk distance test) [32]. In addition, we found that LV systolic function (i.e., LV ejection fraction) was preserved after exercise intervention compared with the control group [32].

Recently, a series of crossover controlled trials conducted at the Hospital for Sick Children and McMaster Children’s Hospital (Toronto and Hamilton, respectively, ON, Canada) assessed exercise intervention effects on survivors of childhood brain tumors who had been treated with cranial radiation (time elapsed since the end of treatment ranging from 1 to 10 years) [48,58,111,112]. The authors found that tailored aerobic exercise intervention might provide benefits on brain volume and structure, physical function and fitness among this population [48,58,111,112]. Thus, the authors suggested the use of exercise as a means to promote cognitive recovery and improve brain function in survivors of childhood brain tumors. Another study conducted in long-term CCS analyzed the effects of a 24-week exercise intervention on vascular endothelial function [47]. The authors used a crossover study design and found that two markers of endothelial function, the delta diameter and flow mediated dilation of the brachial artery, improved with exercise training [47].

Some systematic reviews have been published in recent years including several of the aforementioned articles. For instance, our research group aimed to summarize evidence on the effects of physical exercise interventions in CCS who had completed cancer therapy [113]. Twelve studies were included in the systematic review, and we concluded that, although more research is warranted (especially using RCT designs), exercise interventions appear as a safe and effective option for the improvement of health-related markers in CCS (i.e., vascular endothelial function, PA levels, markers of central adiposity such as waist circumference and waist-to-hip ratio, and brain volume and structure) [113]. Another systematic review aimed to evaluate the evidence on the effectiveness of exercise interventions on PA levels and health behaviors among CCS [114]. On the basis of the eight included RCTs, the authors stated that electronic and mobile health interventions (also known as *eHealth* and *mHealth*, respectively) would be an important strategy to promote PA among CCS, while an education-based approach seemed to be ineffective [114]. A third systematic review focused on exercise interventions for CCS of brain tumors [115]. The authors included five studies, which primarily implemented either an aerobic exercise or an active video-gaming intervention. Although the evidence is still preliminary, in essence, exercise showed promise in improving multiple endpoints (i.e., neural, motor, CRF and, although inconsistent, cognitive endpoints) in children who had been treated for brain cancer [115]. Another recent systematic review addressed the effects of exercise and motor interventions on PA and motor outcomes in CCS of ALL both during and after treatment [116]. The review, which included 19 studies (eight RCTs and two CCTs) showed that exercise improves several health-related endpoints (i.e., fatigue, physical performance, PA levels and coordination) both during and after chemotherapy [116].

Recently, in 2020, an RCT applying a home-based intervention in ALL survivors who were in complete remission for a minimum of one year and had completed cancer treatment was published [40]. The main finding was an improvement of VO_2peak_ in the exercise group compared with the control arm [40]. Finally, Krull et al. evaluated the feasibility and effects of a 24-week strength training intervention with and without protein supplementation on muscle mass and strength among adult CCS [49]. In this double-blind placebo-controlled trial, strength exercise training combined with protein supplementation did not prove to be more effective for increasing total muscle mass and strength than exercise alone [49]. Information on relevant studies analyzing the effects of exercise intervention on CCS after treatment is summarized in Table 3.

### Ongoing Studies

A CCT that started in February 2020 and is being conducted at the Memorial Sloan Kettering Cancer Center (NCT04266080; New York, NY, USA) aims at assessing the effects of a game-based exercise intervention in 60 CCS. The study is expected to be completed in February 2022. In turn, an RCT evaluating a 12-week PA intervention to improve patient-reported outcomes and physical function started in December 2021 and aims to enroll 40 adolescents and young adults who are CCS (NCT04947709; estimated date of study completion, September 2022). Furthermore, an RCT conducted at the St. Jude Children’s Research Hospital (Memphis, TN, USA) aims to enroll 160 adult CCS in order to assess the effects of a tailored home-based exercise intervention (NCT04714840). This study is expected to be completed in December 2023. CRF, cardiovascular function, markers of pulmonary, musculoskeletal, and neurosensory function, and mental health are the main outcomes. Another ongoing study is JUMP (NCT05086354), a single-group feasibility study that aims at assessing the effects of an intervention with fast movements through a jumping rope on balance, coordination, movement speed, and movement agility in childhood ALL survivors (aged 6 to 17 years) who have completed medical treatment within the past five years (1 to 60 months). This study started in March 2018 and is expected to be completed in December 2021.

## 5. Future Perspectives

Studies on the effects of exercise on CCS have used different designs and have applied mostly a supervised training protocol (i.e., in ~two thirds of the studies shown in Table 1, Table 2 and Table 3), followed by mixed (supervised and home-based) and home-based approaches (20% and ~13% of total, respectively). While there is more controversy on the effects of home-based exercise [86,87], evidence seems to support the effectiveness of supervised exercise [33,34,44,91]. Therefore, we suggest that exercise interventions should be supervised, at least in the early stages of the intervention, with the aim of inducing positive adaptations and familiarizing children with the exercises while ensuring safety of the intervention (e.g., acquisition of a good technique for weight training exercises). However, more studies are needed to corroborate that supervised exercise promotes greater benefits than a home-based unsupervised approach. More research is also needed to determine the potential mechanisms whereby exercise training exerts its beneficial effects in CCS. In addition, new RCTs are needed to analyze the effects of exercise training interventions, with the development of a core-set of outcomes in pediatric oncology exercise research. Finally, efforts to individualize exercise prescriptions are required to maximize responsiveness in CCS.

There is accumulating evidence that a healthy lifestyle including regular aerobic PA (e.g., brisk walking), as well as high levels of CRF, are associated with a lower risk of several types of adult cancer [117]. Yet, whether regular PA and/or high CRF can impact pediatric tumor development remains to be determined, at both the preclinical and clinical level. In this regard, the biology of pediatric tumors differs considerably from that of adult tumors. Overall, the former are characterized by a different (and usually lower mutational) burden, an embryonal (or very early) origin in many cases, a dysregulation of developmental pathways, a smaller contribution of environmental factors (which might minimize an eventual anti-cancer benefit of exercise), and tumor development in the context of an immune system that is not yet fully developed/mature [118].

## 6. Conclusions

**Figure 2:**
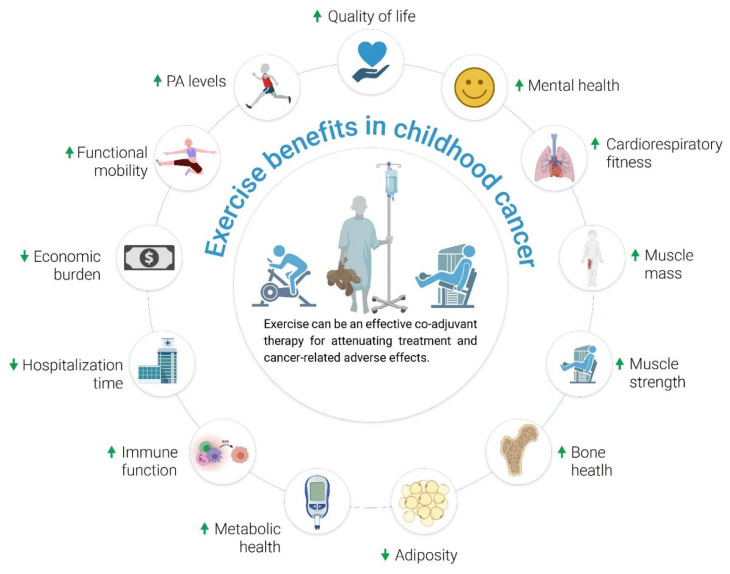
Potential exercise benefits in childhood cancer. Source: self-elaboration. Of note, strong evidence (e.g., based on meta-analyses) is still needed for some of the potential benefits, such as a decrease in hospitalization time or an actual decrease in adiposity (including central adiposity).

To the best of our knowledge, this is the first historical review summarizing, through an analysis of temporal progression, the main milestones in the field of exercise and pediatric oncology. Over the last two decades, the evidence—albeit still sometimes preliminary or emerging—has shown that there seems to be no reason to avoid exercise in CCS, even during the most aggressive phases of cancer treatment (e.g., HSCT). Overall, exercise or regular PA in general is to be considered as a (i) safe and (ii) overall beneficial coadjuvant strategy to attenuate cancer-related adverse effects, with no major contraindications, at least with regard to the different types of pediatric cancers (i.e., in the absence of comorbidities other than cancer per se) (Figure 2). Recently, pediatric oncology exercise guidelines and a recommendation statement have been published [119]. Using the Delphi technique, a panel of experts agreed that movement is important for all children and adolescents affected by cancer and that an exercise professional is recommended, who must consider the patient’s age, their type of cancer, the setting, treatment-related considerations, and individual factors (e.g., previous experience, preferences) [119]. However, these statements are generic in nature and more research is still required to provide specific guidance on the frequency, intensity, duration, and type of PA for this population.

Pediatric exercise oncology is an interesting and incipient field. The data provided in this review intends to modestly summarize the findings in the field—and honor the invaluable efforts of the researchers involved, all of whom are committed to help CCS live a happy, long life.

## Figures and Tables

**Table 1:** Examples of relevant studies analyzing the effects of exercise intervention during childhood cancer treatment.

Study	Study Design	Sample (with Age Expressed as Mean and/or Range)	Primary Cancer	Timing of the Study	Exercise Intervention	Safety	Adherence	Endpoints	Main Results
Braam et al. [88]	RCT	EXP: *N* = 30 (14 female), 13 years	Different types of cancer	During or within the first year of post-cancer treatment	-**Type:** strength + aerobic training -**Intensity:** N/R -**Frequency**: 2 days/week -**Session length**: 45 min per session -**Duration**: 12 weeks -**Supervised**: Yes	N/R	67% attended all exercise sessions (a total of 24 sessions), and the remainder completed an average of 18 sessions (range 10–23)	-**CRF** (VO_2peak_) -**Muscle strength** (handgrip) -**HRQoL** (PedsQL; EQ-5D-Y) -**Cost**-**effectiveness of the intervention**	No major training effect
		CT: *N* = 38 (17 female), 13 years							
Cox et al. [87]	RCT	EXP: *N* = 36 (19 female), 4–18 years	ALL	During treatment (ALL therapy started within the previous 10 days)	-**Type:** strength + aerobic training -**Intensity:** N/R -**Frequency**: 5 days/week -**Session length**: 30 min per session -**Duration**: 2.5 years -**Supervised**: Mixed ^a^	N/R	N/R	-**CRF** (6MWD) -**Muscle strength** (handgrip, knee extension and ankle dorsiflexion—all assessed with a dynamometer) -**Bone mineral density** (DXA) -**BMI** -**HRQoL** (Child Health Questionnaire) -**Ankle dorsiflexion passive ROM** (goniometer) -**PA levels** (accelerometer) -**Motor performance** (BOTSF-2)	No major training effect
		CT: *N* = 41 (18 female), 4–18 years							
Dijk-Lokkart et al. [55]	RCT	EXP: *N* = 30 (14 female), 13 years	Different types of cancer	During or within the first year of post-cancer treatment	-**Type:** strength + aerobic training -**Intensity:** N/R -**Frequency**: 2 days/week -**Session length**: 45 min per session -**Duration**: 12 weeks -**Supervised**: Yes	N/R	Same as above	-**HRQoL** (PedsQL 4.0 generic core scale and PedsQL 3.0 cancer module) -**Fatigue** (18-item PedsQL Multidimensional Fatigue Scale-Acute Version) -**Behavioral problems** (Child Behavior Checklist) -**Mental health** (Children’s Depression Inventory) -**Self**-**perception** (the Dutch versions of the Self Perception Profile for children and adolescents)	↓ parent-reported pain and procedural anxiety with training
		CT: *N* = 38 (17 female), 13 years							
Fiuza-Luces et al. [91]	RCT	EXP: *N* = 24 (7 female), 10 (4–16) years	Different types of cancer	Pre- and post-assessment at the start and end of neoadjuvant chemotherapy, respectively	-**Type**: strength + aerobic training -**Intensity**: 60–70% of HR_max_ (aerobic training) and N/R (strength training) -**Frequency**: 3 days/week -**Session length**: ~60–70 min -**Duration**: 19 weeks on average (range: 9–41) -**Supervised**: Yes	No adverse events noted	68% (SEM = 4%)	-**Muscle strength** (5-RM seated bench, row, and leg press) machines -**CRF** (arm ergometer or treadmill) -**BMI** -**Functional mobility** (TUG and TUDS) -**PA levels** (accelerometer) -**HRQoL** (PedsQL 3.0 cancer module)	↑ muscle strength with training, with these gains partially retained after subsequent 4-month training cessation
		CT: *N* = 25 (7 female), 11 (5–17) years							
Fiuza-Luces et al. [92]	RCT	EXP: *N* = 9 (2 female), 11 years	Different types of cancer	Pre- and post-assessment at the start and end of neoadjuvant chemotherapy, respectively	-**Type**: strength + aerobic training -**Intensity**: 60–70% of maximum heart rate (aerobic training) and N/R (strength training) -**Frequency**: 3 days/week -**Session length**: ~60–70 min -**Duration**: 17 weeks on average (SD = 5 weeks) -**Supervised**: Yes	Same as above (subset of participants of the same trial)	70% on average (SD = 13%)	-**Immune function** (blood samples) -**Inflammation markers** (blood samples) -**PA levels** (accelerometer)	No major training effect
		CT: *N* = 11 (5 female), 12 years							
Hartman et al. [86]	RCT	EXP: *N* = 25 (11 female), 5 (1–16) years	ALL	During treatment (treatment stage not specified)	-**Type**: strength + aerobic training -**Intensity**: N/R -**Frequency**: 7 days/week -**Session length**: N/R -**Duration**: 2 years -**Supervised**: No	29% in the EXP and 12% in the CT sustained fractures, although not necessarily related to the intervention	11% of children exercised daily, 37% > once a week, 16% once weekly, 36% < once a week	-**BMI** -**Bone mineral density, lean mass, and % body fat** (DXA) -**Passive ankle dorsiflexion ROM** (goniometer) -**Motor performance** (BSID-II or Dutch version of the Movement Assessment Battery for Children)	No major training effects
		CT: *N* = 26 (10 female), 6 (2–17) years							
Marchese et al. [50]	RCT	EXP: *N* = 13 (5 female), 7 (4–11) years	ALL	During maintenance treatment	-**Type**: strength + aerobic training -**Intensity**: N/R -**Frequency**: 3 days/week (strength training) and 7 days/week (aerobic training) -**Session length**: 20–60 min -**Duration**: 4 months -**Supervised**: Mixed ^a^	No adverse events noted	N/R	-**Ankle dorsiflexion active ROM** (goniometer) -**Muscle strength** (handgrip) -**Functional mobility** (TUDS) -**CRF** (9MWD) -**HRQoL** (PedsQL version 3.0)	↑ ROM and knee extension strength with training
		CT: *N* = 15 (3 female), 8 (5–16) years							
Morales et al. [34]	Non-RCT	EXP: *N* = 68 (27 female), 11 (4–18) years	Different types of cancer	During the neoadjuvant treatment for solid tumors or during intensive chemotherapy treatment for leukemias	-**Type**: strength + aerobic training -**Intensity**: 65–80% HRR (aerobic training) and gradual increments of 5–10% (strength training) -**Frequency**: 2–3 days/week -**Session length**: ~60–70 min -**Duration**: median of 22 weeks (range: 14–28) -**Supervised**: Yes	No adverse events noted	N/R	-**LV function** (echocardiography) -**Mortality, relapse, and metastasis** (medical reports) -**Length and costs of hospitalization** (medical reports) -**Leukocyte and platelet counts, hemoglobin, and glucose concentration** (blood samples) -**BMI** -**BP**	↓ days and economic cost of hospitalization with training Preserved LV function with training (vs. ↓ in control group)
		CT: *N* = 101 (63 female), 11 (4–18) years							
Moyer-Mileur et al. [38]	RCT	EXP: *N* = 6 (3 female), 7 (4–10) years	ALL	During maintenance treatment	-**Type**: strength + aerobic training -**Intensity**: N/R -**Frequency**: ≥3 days/week -**Session length**: 15–20 min per session -**Duration**: 12 months -**Supervised**: No	N/R	N/R	-**PA levels** (ACTIVITYGRAM Questionnaire and pedometer) -**CRF** (20-m shuttle run test) -**Muscle strength** (push-ups) -**Flexibility** (sit and reach test) -**BMI** -**Muscle mass** (computed tomography)	↑ CRF and PA levels with training
		CT: *N* = 7 (3 female), 6 (4–10) years							
Nielsen et al. [44]	Non-RCT	EXP: *N* = 120 (45 female), 11 years	Different types of cancer	During treatment (treatment stage not specified)	-**Type**: strength + aerobic training -**Intensity**: 69% estimated HR_max_ (mean reported by participants) -**Frequency**: 3 days/week -**Session length**: 5–30 (individually designed activities) and 30–120 (group sessions) minutes per session, respectively -**Duration**: 6 months -**Supervised**: Yes	Six minor events with no complications (i.e., minor bruising, nose bleeding, and fainting) occurred during the intervention.	N/R	-**CRF** (VO_2peak_) -**Muscle strength** (sit to stand test and handgrip) -**Balance** (Flamingo test) -**Functional mobility** (TUG)	Intervention was safe ↑ CRF with training
		CT: *N* = 50 (23 female), 11 years							
San Juan et al. [37]	Non-controlled trial (quasi-experimental design, i.e., participants also assessed 20 weeks after training cessation)	EXP: *N* = 7 (3 female), 5 (4–7) years	ALL	During maintenance treatment	-**Type**: aerobic and strength training -**Intensity**: 50–≥70% HR_max_ (aerobic training) and N/R (strength training) -**Frequency**: 3 days/week -**Session length**: 90–120 min per session -**Duration**: 16 weeks -**Supervised**: Yes	No adverse events noted	>85%	-**CRF** (VO_2peak_ and VO_2peak_ at VT) -**Muscle strength** (6-RM seated bench, row, and leg press) machines -**Functional mobility** (TUDS, TUG) -**Ankle dorsiflexion active and passive ROM** (goniometer) -**HRQoL** (CHIP-CE/CRF)	↑ CRF, muscle strength and functional mobility with training CRF gains partly retained despite training cessation
		No CT							
Saultier et al. [46]	RCT	EXP: *N* = 41 (18 female), 11 years	Different types of cancer	Treatment stage not specified	-**Type**: strength + aerobic training -**Intensity**: 60–70% HR_max_ -**Frequency**: N/R -**Session length**: 30–90 (strength, balance and proprioception training) and 90–240 (e.g., dance, basketball, badminton, yoga, skiing, swimming, paddling) minutes per session, respectively -**Duration**: 6 months -**Supervised**: No	No adverse events noted	N/R	-**CRF** (6MWD) -**Flexibility** (sit and reach test) -**Muscle strength** (sit to stand test, medicine-ball launch, and trunk and abdominal muscle endurance) -**Balance** (Flamingo test) -**BMI** -**Lean and fat mass** (impedance meter) -**Self**-**esteem** (PSI-VSF) -**HRQoL** (VSP)	Intervention was safe ↑ CRF, flexibility, muscle strength, balance with training
		CT: *N* = 39 (16 female), 11 years							
Shore and Shepard [43]	Non-RCT	EXP: *N* = 3 (N/R female), 14 years	LLA and other types of cancer	Having completed the treatment or under active treatment	-**Type**: aerobic training -**Intensity**: 70–85% HR_max_-**Frequency**: 3 days/week -**Session length**: 30 min per session -**Duration**: 12 weeks -**Supervised**: Mixed ^a^	N/R	N/R	-**CRF** (VO_2peak_) -**Leukocyte and lymphocyte counts and hemoglobin** (blood samples) -**Immune function** (blood samples) -**Mental health** (Piers-Harris Self-Concept Scale)	↑ CRF and mental health with training ↓ (albeit ‘not clinically significant’) immune function with training
		CT: *N* = 3 (N/R female), 13 years							
Stössel et al. [45]	RCT	EXP: *N* = 16 (6 female), 11 (4–17) years	Different types of cancer	During treatment (treatment stage not specified)	-**Type**: strength + aerobic training -**Intensity**: 60–75% of estimated HR_max_ or a score of 12 to 13 on the Borg scale RPE 6–20 -**Frequency**: 3 days/week -**Session length**: 45–60 min per session -**Duration**: 3 months -**Supervised**: Mixed ^a^	Safe except for some falls with no injuries and light-to-moderate muscle soreness in some participants.	N/R	-**CRF** (6MWD) -**Muscle strength** (handgrip and knee strength measured with dynamometer) -**BMI** -**phA** (impedance meter) -**Fatigue** (PedsQL 3.0 Multidimensional Fatigue Scale) -**HRQoL** (KINDL) -**PA levels** (MoMo questionnaire)	↑ CRF, lower limb muscle strength, and PA levels with training ↓ patients’ self-reported fatigue with training
		CT: *N* = 17 (7 female), 11 (5–18) years							
Tanir and Kuguoglu [39]	RCT	EXP: *N* = 19 (4 female), 10 (8–12) years	ALL	On remission after +1 year of diagnosis	-**Type**: strength + aerobic training -**Intensity**: N/R -**Frequency**: 3 days/week -**Session length**: N/R -**Duration**: 3 months -**Supervised**: No	N/R	N/R	-**CRF** (9MWD) -**Muscle strength** (dynamometer) -**Functional mobility** (TUDS, TUG) -**HRQoL** (PedsQL 4.0 and PedsQL 3.0 cancer module) -**ROM** (goniometer)	↑ CRF, muscle strength, and functional mobility with training ↓ worry scores with training
		CT: *N* = 21 (9 female), 11 (8–12) years							
Winter et al. [54]	Non-RCT	EXP: *N* = 16 (9 female), 14 years	Bone tumors	During treatment (treatment stage not specified)	-**Type**: strength + aerobic training -**Intensity**: N/R -**Frequency**: Once a day -**Session length**: 30–60 min per session -**Duration**: 3 days -**Supervised**: Yes	No adverse events noted	58% All patients participated in ≥40% of the sessions	-**PA levels** (accelerometer)	↑ PA levels with training
		CT: *N* = 15 (6 female), 14 years							

Abbreviations: ALL, acute lymphoblastic leukemia; BMI, body mass index; BOT-2, The Bruininks-Oseretsky Test of Motor Proficiency 2nd Edition; BP, blood pressure; BSID-II, Dutch Bayley Scales of Infant Development; CHIP-CE/CRF, the Child’s Health and Illness Profile—Child Edition; CRF, cardiorespiratory fitness; CT, control group; DXA, dual-energy X-ray absorptiometry; EXP, experimental group; EQ-5D-Y, European Quality of Life-5 Dimensions youth version; HR_max_, maximum heart rate; HRQoL, health-related quality of life; HRR, heart rate reserve; LV, left ventricular; N/R, not reported; PA, physical activity; phA, phase angle; PedsQL, Pediatric Quality of Life Inventory; PSI-VSF, Physical Self-Inventory—Very Short Form; RCT, randomized controlled trial; RM, repetition maximum; ROM, range of movement; RPE, rate of perceived exertion; SD, standard deviation; SEM, standard error of the mean; TUG, Timed Up and Go; TUDS, Timed Up and Down Stairs; VO_2peak_, peak oxygen uptake; VSP, “*Vécu et Santé Perçue de l’Adolescent et de l’enfant*” questionnaire; VT, ventilatory threshold; 6MWD, 6-min walk distance; 9MWD, 9-min walk distance. Symbol: ^a^ Supervised + not supervised.

**Table 2:** Examples of relevant studies on the effects of exercise intervention during/after childhood hematopoietic stem cell transplantation (HSCT).

Study	Study Design	Sample (with Age Expressed as Mean and/or Range)	Diagnosis	Main HSCT Characteristics	Exercise Intervention	Safety	Adherence	Endpoints	Main Results
Chamorro-Viña et al. [52]	Non-RCT	EXP: *N* = 7 (2 female), 8 years	Different types of cancer	-**Type**: allogeneic -**Stage**: during HSCT (started at the beginning of the conditioning phase –pretransplant– and lasted until the end of the neutropenic phase) -**Prophylactic therapy**: acyclovir, cyclosporine and methotrexate -**Conditioning regimen**: thiotepa, fludarabine, dexamethasone and busulfan	-**Type**: strength + aerobic training -**Intensity**: 50–70% of the estimated HR_max_ (aerobic training) and light loads (strength training) -**Frequency**: 5 days/week -**Session length**: ~50 min per session -**Duration**: ~3 weeks -**Supervised**: Yes	No adverse events noted	>90%	-**Immune function** (blood samples) -**BMI and body mass** -**Lean and fat mass** (estimated through skinfold measures)	↑ BMI with training Preserved dendritic cell count post-HSCT with training (vs. the control group)
		CT: *N* = 13 (4 female), 7 years							
Chamorro-Viña et al. [53]	RCT	EXP: N = 3 (2 female), 9–17 years	Different types of malignant and nonmalignant conditions	-**Type**: allogeneic -**Stage**: discharged from HSCT no later than day +30 -**Prophylactic therapy**: cyclosporine and methotrexate -**Conditioning regimen**: nonmyeloablative	-**Type**: strength + aerobic training -**Intensity**: 50–70% of the estimated HR_max_ (aerobic training) and 70–80% 1-RM (strength training) -**Frequency**: 3 days/week -**Session length**: 60 min per session -**Duration**: 10 weeks -**Supervised**: Mixed ^a^	No adverse events noted	80%	-**Immune function** (blood samples) -**Muscle strength** (6-RM seated bench, row, and leg press test, respectively) -**Functional mobility** (TUDS, TUG)	↑ CD56^dim^ NK cells with training
		CT: *N* = 3 (3 female), 8–19 years							
Davis et al. [42]	Non-controlled trial	EXP: *N* = 20 (8 female), 17 (11–25) years	Blood cancer	-**Type**: N/R -**Stage**: >1 year post-HSCT. Mean time since HSCT: 8 (2–16) years	-**Type**: strength + aerobic training -**Intensity**: 60–70% of HR_max_ (aerobic training) and 60–80% 1-RM (strength training) -**Frequency**: 2–3 days/week -**Session length**: 45–60 min per session -**Duration**: 6 months -**Supervised**: Yes	N/R	85% participants attended at least twice weekly and 35% participants attended three times weekly	-**CRF** (VO_2peak_, VT, RER, and O_2_ pulse) -**Lean and fat mass and % trunk fat** (DXA) -**BMI** -**HRQoL** (SF-36 and MMQL) -**Glucose and insulin concentrations and HOMA**-**IR** (blood samples) -**Pulmonary function** (FVC and FEV_1_)	↑ VO_2peak_, O_2_ pulse, BMISDS, and SF-36 (general health domain) and MMQL (school domain) with training ↓ insulin concentration and HOMA-IR with training
		No CT							
Morales et al. [33]	Non-RCT	EXP: *N* = 65 (24 female), 11 (5–18) years	Blood cancer	-**Type**: autologous and allogeneic -**Stage**: during HSCT (at the beginning of the conditioning phase—pretransplant—and lasted until the end of the neutropenic phase) -**Prophylactic therapy**: cyclosporine and methylprednisolone -**Conditioning regimen**: myeloablative or nonmyeloablative	-**Type**: strength + aerobic training -**Intensity**: 65–80% of HRR (aerobic training) and N/R (strength training) -**Frequency**: 5 days/week -**Session length**: ~60 min per session -**Duration**: ~3 weeks -**Supervised**: Yes	No adverse events noted	N/R	-**Mortality, risk of GvHD or new HSCT** (medical reports) -**Engraftment kinetics, supportive care, toxicity profile and infections** (medical reports) -**Immune reconstitution** (blood samples)	Intervention was safe and well tolerated ↓ number of total and viral infections after allogenic HSCT with training
		CT: *N* = 53 (20 female), 10 (4–18) years							
San Juan et al. [41]	Non-RCT	EXP: *N* = 8 (4 female), 11 (8–16) years	ALL and AML	-**Type**: haploidentical and allogeneic -**Stage**: <12 months post-HSCT. Mean time since HSCT: 9 (2–12) months -**Prophylactic therapy**: mycophenolate and methylprednisolone	-**Type**: strength + aerobic training -**Intensity**: 50–≥70% of the estimated HR_max_ (aerobic training) and N/R (strength training) -**Frequency**: 3 days/week -**Session length**: 90–120 min per session -**Duration**: 8 weeks -**Supervised**: Yes	No adverse events noted	>70% in 7 children and 50% in 1 child	-**CRF** (VO_2peak_ and VT) -**Muscle strength** (6-RM seated bench, row, and leg press test, respectively) -**Functional mobility** (TUDS, TUG) -**HRQoL** (CHIP-CE/CRF and CHIP-PE/AE)	↑ VO_2peak_, muscle strength, TUDS and HRQoL
		CT (healthy children): *N* = 8 (4 female), 11 years							
Senn-Malashonak et al. [51]	RCT	EXP: *N* = 35 (10 female), 11 (5–17) years	Different types of cancer	-**Type**: autologous and allogeneic -**Stage**: during HSCT (from admission to discharge) -**Conditioning regimen**: myeloablative or reduced intensity conditioning	-**Type**: strength + aerobic training -**Intensity**: 12–14 on the Borg’s 6–20 RPE scale -**Frequency**: 5 days/week -**Session length**: 30–60 min per session -**Duration**: N/R -**Supervised**: Yes	No adverse events noted	94% (range = 63–100%)	-**CRF** (6MWD, VO_2peak_ and VT) -**Muscle strength** (isometric maximal knee extension strength and handgrip) -**HRQoL** (KINDL) -**Immune reconstitution** (blood samples) -**Leukocyte, granulocyte and thrombocyte counts** (blood samples) -**Day of engraftment and complications** (medical reports)	Intervention was safe Preserved 6MWD, knee extension strength and handgrip with training (vs. ↓ in the control group) ↓ HRQoL in both groups
		CT: *N* = 35 (12 female), 12 (6–18) years							
Yildiz Kabak et al. [103]	Non-RCT	EXP: *N* = 11 (N/R female), 9 (4–15) years	Different types of malignant and nonmalignant conditions	-**Type**: autologous and allogeneic -**Stage**: during HSCT (from admission to discharge) -**Conditioning regimen**: myeloablative or reduced intensity conditioning	-**Type**: strength + aerobic training -**Intensity**: 10–13 on the Borg scale RPE 6–20 -**Frequency**: 5 days/week -**Session length**: 20–40 min per session -**Duration**: 41 days (average stay in the hospital) -**Supervised**: Yes	N/R	82%	-**CRF** (6MWD) -**Muscle strength** (30-s chair-stand test and handgrip) -**Functional mobility** (TUDS, TUG, time needed to stand up from bed rest exam, and WeeFIM) -**BMI**	↑ 6MWD as well as performance in most functional mobility tests (30-s chair-stand, TUDS, TUG, and time needed to stand up from bed rest) with exercise intervention
		CT: *N* = 11 (N/R female), 7 (4–11) years							

Abbreviations: BMI, body mass index; BMISDS, body mass index standard deviation score; CHIP-CE/CRF, Child’s Health and Illness Profile-Child Edition; CHIP-PE/AE, Child’s Health and Illness Profile-Adolescent Edition; CT, control group; DXA, dual energy X-ray absorptiometry; EXP, experimental group; FEV_1_, forced expiratory volume over the first second; FVC, forced vital capacity; GvHD, graft versus host disease; HOMA-IR, homeostatic model assessment of insulin resistance; HRQoL, health-related quality of life; HRR, heart rate reserve; MMQL, Minneapolis-Manchester Quality of Life Instrument; NK, natural killer; RER, respiratory exchange ratio; RM, repetition maximum; RPE, rate of perceived exertion; SF-36, 36-Item Short Form Health Survey; VO_2peak_, peak oxygen uptake; VT, ventilatory threshold; WeeFIM, functional independent measure for children; 6MWD, 6-min walk distance. Symbols: ^a^ Supervised + not supervised.

**Table 3:** Examples of relevant studies analyzing the effects of exercise intervention after childhood cancer treatment.

Study	Study Design	Sample (with Age Expressed as Mean and/or Range)	Main Cancer Characteristics	Exercise Intervention	Safety	Adherence	Endpoints	Main Results
Cox et al. [48]	Crossover controlled trial	EXP: *N* = 25 (11 female), 12 (7–17) years	Type of cancer: brain tumor Mean age at diagnosis: 6 (2–9) years Mean time since diagnosis: 5 (1–10) years Time since treatment: 1–10 years Time of remission: N/R Treatment: surgery and/or CRT and/or chemotherapy	-**Type**: aerobic training -**Intensity**: ~80% HR_max_ -**Frequency**: 3–4 days/week -**Session length**: 90 min (group setting) and 30 min per session (home setting) -**Duration**: 12 weeks -**Supervised**: Yes	N/R	90%	-**Cognitive function** (Go task and Go/No-Go task during magnetoencephalography, functional connectivity assessed through electroencephalography, FSIQ) -**Neurological function** (cerebellar signs such as ataxia, dysmetria, and dysdiadochokinesia, motor deficit, cranial nerve deficit, and visual impairment) -**CRF** (6MWD) -**Functional mobility** (BOT-2)	↑ cognitive function (i.e., increased response accuracy and functional mechanisms under task load) and CRF
		CT: *N* = 25 (11 female), 12 (7–17) years						
Järvelä et al. [35]	Non-controlled trial	EXP: *N* = 17 (9 female), 22 (16–30) years	Type of cancer: ALL Mean age at diagnosis: 5 (2–13) years Median time since diagnosis: 16 (11–21) years Time since treatment: N/R Time of remission: first remission Treatment: anthracyclines (median: 240 mg/m^2^) and/or CRT	-**Type**: aerobic and strength training -**Intensity**: N/R -**Frequency**: 3–4 days/week -**Session length**: 30 min per session (aerobic training) -**Duration**: 16 weeks -**Supervised**: No	No adverse events noted	N/R	-**CRF** (VO_2peak_) -**LV function** (echocardiography) -**Dynamic muscle strength** (sit-up and back tests, a 30-s full squat test, and lifting weights) -**Muscle power of the lower extremities** (vertical squat jump) -**Maximum isometric handgrip strength** (dynamometer) -**BMI, weight, waist circumference, and waist**-**to**-**hip ratio** -**% fat mass** (skinfold) -**BP** -**PA levels** (questionnaire) -**Plasma glucose, insulin, HOMA**-**IR, HDL, LDL and total cholesterol, and triglyceride concentrations** (blood analysis)	↑ CRF, sit-up and back test and full squat test, and PA levels with training ↓ waist circumference, waist-to-hip ratio, % fat mass, diastolic BP, plasma insulin, and HOMA-IR with training Preserved LV function with training
		No CT						
Järvelä et al. [36]	Non-controlled trial	EXP: *N* = 17 (9 female), 23 (17–30) years	Type of cancer: ALL Mean age at diagnosis: 5 (2–13) years Median time since diagnosis: 16 (11–21) years Time since treatment: N/R Time of remission: first remission Treatment: anthracyclines (median: 240 mg/m^2^) and/or CRT	-**Type**: aerobic and strength training -**Intensity**: N/R -**Frequency**: 3–4 days/week -**Session length**: 30 min per session (aerobic training) -**Duration**: 12 weeks -**Supervised**: No	N/R	N/R	-**LV structure and function** (echocardiography)	↑ early diastolic mitral inflow velocity, peak circumferential strain rate and diastolic strain rate, respectively, at post-intervention Preserved LV function and LV end-systolic and end-diastolic dimensions and volumes, intraventricular septum thickness, and LV posterior wall thickness or mass at post-intervention
		No CT						
Krull et al. [49]	RCT	EXP (strength training + protein supplementation): *N* = 29 (13 female), 33 (21–44) years	Type of cancer: different types of cancer Median age at diagnosis: ~8 (0–20) years Median time since diagnosis: ~23 (10–44) years Time since treatment: N/R Time of remission: N/R Treatment: surgery and/or CRT and/or chemotherapy	-**Type**: strength training -**Intensity**: 60–80% 1-RM -**Frequency**: 3 days/week -**Session length**: N/R -**Duration**: 24 weeks -**Supervised**: Yes	Minor adverse events including knee pain, muscle soreness, nausea, pain and anxiety. A myocardial infarction occurred, although not during training sessions.	75% (strength training + protein supplementation) and 67% (only strength training)	-**Muscle strength** (1-RM) -**Lean mass** (DXA) -**Functional mobility** (10-m walk test) -**HRQoL** (SF-36) -**PA levels** (accelerometer)	Intervention was safe ↑ muscle strength and lean mass both with training only and with training + supplementation
		CT (only strength training): *N* = 38 (19 female), 34 (21–45) years						
Long et al. [47]	Crossover controlled trial	EXP: *N* = 13 (7 female), 19 (16–23) years	Type of cancer: brain tumor, ALL and rhabdomyosarcoma Median age at diagnosis: 3 (0–10) years Median time since diagnosis: 15 (7–22) years Median time since treatment: 13 (7–21) years Time of remission: N/R Treatment: surgery and/or HSCT and/or chemotherapy and/or radiotherapy	-**Type**: aerobic and strength training -**Intensity**: ~60% HR_max_ (aerobic training) and ~50–60% 1-RM (strength training) -**Frequency**: 3 days/week -**Session length**: 90 min per session -**Duration**: 24 weeks -**Supervised**: Yes	No adverse events noted	N/R	-**Dynamic muscle strength** (*latissimus dorsi* pull-down and biceps curl) -**Muscular endurance** (squats, sit-ups and push-ups) -**CRF** (submaximal and VO_2peak_) -**Lean and fat mass, peripheral and visceral adipose tissue** (DXA) -**Weight and BMI** -**HR and BP** -**FMD and delta diameter of the brachial artery** (vascular ultrasound) -**PA levels** (accelerometer)	↑ biceps curl strength, V_E_, RER, relative VO_2peak_, delta diameter, FMD, and breaks in sedentary time with training
		CT: *N* = 13 (7 female), 19 (16–23) years						
Manchola-Gonzalez et al. [40]	RCT	EXP: *N* = 12 (12 female), 12 (7–17) years	Type of cancer: ALL Age at diagnosis: N/R Time since diagnosis: N/R Time since treatment: N/R Time of remission: >1 year Treatment: N/R	-**Type**: aerobic and strength training -**Intensity**: 50–80% HR_max_ -**Frequency**: 3 days/week -**Session length**: 15–30 min per session (aerobic training) -**Duration**: 16 weeks -**Supervised**: No	No adverse events noted	75%	-**CRF** (VO_2peak_, maximal load, O_2_ pulse, VCO_2_, V_E_, RER and AT) -**Muscle strength** (handgrip) -**Flexibility** (sit-and-reach test) -**Functional mobility** (TUDS and TUG) -**BMI** -**PA levels** (PAQ-A)	↑ VO_2peak_ with training
		CT: *N* = 12 (12 female), 11 (7–17) years						
Piscione et al. [111]	Crossover controlled trial	EXP: *N* = 28 (12 female), 12 (8–17) years	Type of cancer: brain tumor Mean age at diagnosis: 6 (2–9) years Mean time since diagnosis: 5 (1–10) years Time since treatment: 1–10 years Time of remission: N/R Treatment: surgery and/or CRT and/or chemotherapy	-**Type**: aerobic training -**Intensity**: ~80% HR_max_ -**Frequency**: 2–5 days/week -**Session length**: 90 (group setting) and 30 min per session (home setting) -**Duration**: 12 weeks -**Supervised**: Yes	No adverse events noted	84%	-**CRF** (VO_2peak_ and pro-rated work rate) -**Functional mobility** (BOT-2)	↑ pro-rated work rate and bilateral coordination with training
		CT: *N* = 28 (12 female), 12 (8–17) years						
Riggs et al. [112]	Crossover controlled trial	EXP: *N* = 28 (12 female), 12 (8–17) years	Type of cancer: brain tumor Mean age at diagnosis: 6 (2–9) years Mean time since diagnosis: 5 (1–10) years Time since treatment: 1–10 years Time of remission: N/R Treatment: surgery and/or CRT and/or chemotherapy	-**Type**: aerobic training -**Intensity**: ~80% HR_max_ -**Frequency**: 2–5 days/week -**Session length**: 90 min (group setting) and 30 min per session (home setting) -**Duration**: 12 weeks -**Supervised**: Yes	No adverse events noted	84%	-**CRF** (6MWD) -**Attention, processing speed and short**-**term memory** (CANTAB) -**White matter architecture and hippocampal volume** (MRI)	Intervention was safe ↓ reaction time with training ↑ white matter fractional anisotropy and hippocampal volume with training
		CT: *N* = 28 (12 female), 12 (8–17) years						
Sharkey et al. [107]	Non-controlled trial	EXP: *N* = 10 (5 female), 19 years	Type of cancer: different types of cancer Mean age at diagnosis: 8 years Time since diagnosis: N/R Time since treatment: N/R Time of remission: in complete remission Treatment: anthracyclines (mean: 349 mg/m^2^) and/or radiotherapy	-**Type**: aerobic training -**Intensity**: 60–80% HR_max_ -**Frequency**: 2–3 days/week -**Session length**: 45–60 min per session -**Duration**: 12 weeks -**Supervised**: Mixed ^a^	N/R	All patients attended 15–18 sessions out of 24	-**CRF** (VO_2peak_, exercise time, HR_max_, peak cardiac index, peak stroke volume index, minimal systemic vascular resistance index, and AT) -**Pulmonary function** (FVC and FEV_1_) -**% body fat** (N/R) -**BP**	↑ total exercise time with training
		No CT						
Szulc-Lerch et al. [58]	Crossover controlled trial	EXP: *N* = 28 (12 female), 12 (8–17) years	Type of cancer: brain tumor Mean age at diagnosis: 6 (2–9) years Mean time since diagnosis: 5 (1–10) years Time since treatment: 1–10 years Time of remission: N/R Treatment: surgery and/or CRT and/or chemotherapy	-**Type**: aerobic training -**Intensity**: ~80% HR_max_ -**Frequency**: 2–5 days/week -**Session length**: 90 min (group setting) and 30 min per session (home setting) -**Duration**: 12 weeks -**Supervised**: Yes	No adverse events noted	84%	-**Cortical thickness and brain volume** (MRI)	↑ cortical thickness and white matter volume with training
		CT: *N* = 28 (12 female), 12 (8–17) years						
Takken et al. [108]	Non-controlled trial	EXP: *N* = 4 (1 female), ~9 (6–14) years	Type of cancer: ALL Age at diagnosis: N/R Time since diagnosis: N/R Time since treatment: 1–3 years Time of remission: in complete remission Treatment: chemotherapy	-**Type**: aerobic and strength training -**Intensity**: 66% to >90% of HR_max_ -**Frequency**: 4 days/week -**Session length**: 45 min per session -**Duration**: 12 weeks -**Supervised**: Mixed ^a^	Minor symptoms including headache, muscle soreness, fatigue and hyperventilation	N/R	-**CRF** (VO_2peak_) -**Muscle strength** (handgrip) -**Fatigue** (CIS-20) -**BMI, weight** -**Fat mass** (skinfold) -**Functional mobility** (TUDS and TUG)	No major training effect
		No CT						

Abbreviations: ALL, acute lymphoblastic leukemia; AT, anaerobic threshold; BMI, body mass index; BOT-2, The Bruininks-Oseretsky Test of Motor Proficiency 2^nd^ Edition; BP, blood pressure; CANTAB, The Cambridge Neuropsychological Test Automated Battery; CIS-20, checklist individual strength questionnaire; CRF, cardiorespiratory fitness; CRT, cranial radiation therapy; CT, control group; DXA, dual-energy X-ray absorptiometry; EXP, experimental group; FEV_1_, forced expiratory volume over the first second; FMD, flow mediation dilation; FSIQ, full scale intelligence quotient; FVC, forced vital capacity; HDL, high-density lipoprotein; HOMA-IR, homeostasis model assessment of insulin resistance; HR, heart rate; HR_max_, maximum heart rate; HRQoL, health-related quality of life; HSCT, hematopoietic stem cell transplantation; LDL, low-density lipoprotein; LV, left ventricular; MRI, magnetic resonance imaging; N/R: not reported; PA, physical activity; PAQ-A, Physical Activity Questionnaire for Adolescents; RCT, randomized controlled trial; RER, respiratory exchange ratio; RM, repetition maximum; SF-36, 36-Item Short Form Health Survey; TUDS, timed up and down stairs test; TUG, timed up-and-go test; VCO_2_, output of carbon dioxide; V_E_, minute ventilation; VO_2peak_, peak oxygen uptake; 6MWD, 6-min walk distance. Symbol: ^a^ Supervised + not supervised.
